# A Non-linear Association Between Total Small Vessel Disease Score and Hemorrhagic Transformation After Ischemic Stroke With Atrial Fibrillation and/or Rheumatic Heart Disease

**DOI:** 10.3389/fneur.2019.00769

**Published:** 2019-07-24

**Authors:** Chenchen Wei, Junfeng Liu, Jie Li, Ming Liu

**Affiliations:** ^1^Department of Neurology, West China Hospital, Sichuan University, Chengdu, China; ^2^Department of Neurology, People's Hospital of Deyang City, Deyang, China

**Keywords:** cerebral small vessel disease, hemorrhagic transformation, ischemic stroke, atrial fibrillation, rheumatic heart disease

## Abstract

**Background:** Previous studies have investigated the association between a single marker of cerebral small vessel disease (SVD) and hemorrhagic transformation (HT). However, the effect of the total SVD burden on HT has not been evaluated yet. We aimed to investigate the association between the total SVD score and HT in ischemic stroke patients with atrial fibrillation (AF) and/or rheumatic heart disease (RHD).

**Methods:** Ischemic stroke patients with AF and/or RHD admitted within 7 days after onset were enrolled at two hospitals in China. The total SVD score was based on the presence of lacunes, extensive white matter hyperintensities, cerebral microbleeds, and moderate to severe enlarged perivascular spaces in the basal ganglia. One point was awarded for the presence of each marker, with the total SVD score ranging from 0 to 4 points. HT was assessed based on follow-up imaging scans during hospitalization and was classified according to the radiographic appearance and associated neurological deterioration.

**Results:** Of 207 enrolled patients (mean age, 67.79 years; 58.9% female), 89 (43.0%) developed HT. The distribution of the total SVD score was significantly different between patients with and without HT in the univariate analysis (*p* = 0.04). After adjustment for confounders, a SVD score of 1 was independently associated with an increased risk of HT [odds ratio (OR), 3.23; 95% confidence interval (CI), 1.48–7.04; *p* = 0.003], while a SVD score ≥2 was inversely related to the occurrence of HT (OR, 0.41; 95% CI, 0.19–0.91; *p* = 0.03). These independent associations remained significant in the subgroups of hemorrhagic infarction and asymptomatic HT (all *p* < 0.05).

**Conclusions:** In our study, the relationship between the total SVD score and HT was not linear, since the presence of only one marker of SVD was associated with an increased risk of HT, while the presence of two or more markers of SVD was a potential protective factor for HT. These results indicate the need to take the total SVD score into account, not only a single SVD marker, when assessing the risk of HT. Further studies with larger samples are required to validate these findings.

## Introduction

Stroke is the second leading cause of death worldwide and the leading cause of death in China (1). Cardioembolic stroke, constituting 10–26% of all ischemic strokes in China (2), is the most severe subtype of ischemic stroke with a high in-hospital mortality and disability at discharge (3). Current treatment guidelines recommend anticoagulants, thrombolysis, and endovascular manipulation in eligible patients with cardioembolic stroke (4–6). Despite the recommendations, these treatments are underused due to the concern about the risk of hemorrhagic transformation (HT), a complication of ischemic stroke, especially in China (1). Therefore, identifying the risk factors for HT and understanding the underlying mechanisms would aid the early identification of patients at high risk of HT and facilitate the selection of therapeutic interventions for ischemic stroke.

Cerebral small vessel disease (SVD) is an age-related disorder comprising various pathological processes and affecting the small blood vessels of the brain (7). Four major markers detectable through magnetic resonance imaging (MRI) are associated with SVD: lacunes, white matter hyperintensities (WMHs), cerebral microbleeds (CMBs), and enlarged perivascular spaces (EPVSs) (7, 8). In order to assess the overall burden of SVD on the brain, a total SVD score has been proposed that includes all four MRI markers, rather than only one or two (9, 10). Several studies found that the total SVD score is associated with high risk of recurrent stroke (11), mortality (12), cognitive decline (13), and depression (14) after stroke. Although previous publications investigated the association between cerebral SVD and HT (15–18), most of them focused on a single SVD marker. We are unaware of studies addressing the effect of total SVD burden on HT. Therefore, whether total SVD burden correlates with HT and whether higher SVD burden increases risk of HT are unknown.

In the present study, we aimed to investigate the association between the total SVD score and HT in ischemic stroke patients with atrial fibrillation (AF) and/or rheumatic heart disease (RHD). We selected ischemic stroke patients with AF and/or RHD as the study cohort because they are treated most often with thrombolysis and anticoagulants, and because they show a higher incidence of HT than patients with other subtypes of stroke (19).

## Materials and Methods

### Study Subjects

We enrolled patients with ischemic stroke admitted to West China Hospital of Sichuan University (Chengdu, China) between October 2013 and December 2017, and to the People's Hospital of Deyang City (Deyang, China) between October 2014 and July 2015. The inclusion criteria were (1) patients that experienced ischemic stroke confirmed by brain computed tomography (CT) or MRI who were admitted within 7 days after stroke onset, (2) an initial CT scan on admission and follow-up CT or MRI scans during hospitalization were performed, and (3) patients received an additional diagnosis of AF and/or RHD at discharge. Patients were excluded if the quality of brain scans was poor, making the assessment of SVD difficult, or if the relevant MRI sequences were unavailable, including T1-weighted, T2-weighted, fluid-attenuated inversion recovery (FLAIR), and susceptibility-weighted imaging.

Demographic and clinical information was extracted from medical databases, including age, sex, prior medical history (hypertension, diabetes mellitus, and hyperlipidemia), previous use of antithrombotic agents (i.e., antiplatelets and anticoagulants), smoking, alcohol consumption, stroke severity assessed by the National Institutes of Health Stroke Scale (NIHSS) on admission, blood pressure on admission, laboratory tests at the first visit, and treatments during hospitalization including antiplatelets, anticoagulants, and thrombolysis. Ischemic stroke was classified as cardioembolic, atherothrombotic, lacunar, or uncertain stroke based on the etiological classification defined by the Stroke Prevention in Atrial Fibrillation (SPAF) study (20).

The study was approved by the Biomedical Research Ethics Committee of West China Hospital, Sichuan University, and the protocol followed the local ethics criteria for human research. Written informed consent was obtained from all the participants or their legal representatives.

### Neuroimaging: CT and MRI Scans

Initial CT scans were performed within 24 h after admission, followed by scheduled MRI within 7 days after admission or repeated CT immediately whenever hemorrhage was suspected, such as in the case of headache or neurological deterioration. All CT examinations were performed using 64-section scanners (Siemens, Berlin, Germany) with 7-mm slice thickness. MRI images were obtained with a 1.5-T scanner (Philips, Amsterdam, The Netherlands) at the People's Hospital of Deyang City or a 3-T Siemens scanner at West China Hospital. MRI parameters have been previously described (15). All images were assessed by two experienced researchers who were blinded to clinical information. Disagreement was solved through discussion or advice from a third researcher. Interrater kappa and intrarater reliability for imaging markers were determined. The latter was calculated based on a single researcher's repeat assessments of 50 randomly selected brain scans.

### Definition of HT

HT was defined as hemorrhage within the infarct territory or parenchyma hemorrhage outside the infarct zone that was detected on follow-up CT or MRI during hospitalization, but not on head CT on admission (21). In terms of the radiographic appearance of the hemorrhage, HT was classified into two groups according to the criteria of the European Cooperative Acute Stroke Study II (22): hemorrhagic infarction (HI), when a petechial infarction without space-occupying effect was observed, or parenchymal hemorrhage (PH), when a hemorrhage (coagulum) with mass effect was observed. Regarding the presence of associated neurological deterioration, HT was classified as symptomatic HT when the patients showed an increase of four points or more in the NIHSS score, or as asymptomatic HT when the patients showed no worsening of neurological manifestations (23). For HT, the interrater kappa was 0.92, and the intrarater kappa was 0.96.

### Total SVD Score

The total SVD score comprised four MRI markers of SVD (lacunes, WMHs, CMBs, and EPVS). One point was awarded for the presence of each of the four markers, so scores could range from 0 to 4 (9, 10). Lacunes were defined as round or ovoid lesions with diameters of 3–20 mm, showing cerebrospinal fluid-like hyperintensity on T2-weighted imaging with corresponding hypointense lesions with a hyperintense rim on FLAIR; these lesions were located in the basal ganglia, thalamus, internal or external capsule, or brain stem (8, 9). The presence of one or more lacunes received one point on the SVD score. WMH was defined as the presence of hyperintense lesions on both FLAIR and T2-weighted imaging, in the absence of prominent hypointensity on the T1-weighted imaging (8). WMHs were classified as located either in the periventricular or the deep white matter and were graded with a Fazekas score from 0 to 3 (24). One point on the SVD score was awarded when periventricular WMH was graded 3 and/or deep WMH was graded 2–3. CMB was defined as a homogeneous, circular focal area, with a diameter <10 mm and very low signal intensity on susceptibility-weighted imaging (8). The number and location of CMBs were recorded. One point was awarded if one or more CMBs were present. EPVS was defined as small puncta in the axial section (in the basal ganglia) and lines in the longitudinal section (in the centrum semiovale) <3 mm wide. EPVS appeared hyperintense on T2-weighted imaging and hypointense on T1-weighted imaging and FLAIR, and it was graded from 0 to 4 (25). The presence of moderate to severe basal ganglia EPVS (grades 2–4) was assigned one point on the SVD score. The presence of lacunes, WMHs, CMBs, and EPVS was assessed outside the acute infarct area. The interrater kappa coefficient was 0.85 for lacunes, 0.84 for periventricular WMH, 0.86 for deep WMH, 0.80 for CMBs, and 0.83 for EPVS. The intrarater coefficients were at least 0.87.

### Statistical Analysis

Results are presented as percentages, mean ± standard deviations (SDs), median with interquartile range (IQR), or odds ratios (ORs) with 95% confidence intervals (CIs), as appropriate. We compared the differences in the baseline characteristics between the groups of HT and non-HT using Student's *t*-test or the Mann–Whitney *U*-test for continuous variables and χ^2^ or Fisher's exact test for categorical variables. One-way analysis of variance (ANOVA), χ^2^, or the Kruskal–Wallis test was used to compare results among subgroups with different SVD scores. A multivariate logistic regression model was used to assess the independent association between the total SVD score and HT. The model included variables that were found to be potentially associated with HT in the univariate analyses (*p* < 0.10), as well as age, NIHSS score, blood glucose on admission, etiology of stroke, use of antiplatelets or anticoagulants, and use of thrombolysis, which are considered risk factors for HT (19, 26, 27). For all analyses, a two-sided *p* < 0.05 was considered statistically significant. Statistical analyses were performed using SPSS version 22.0 (IBM, USA).

## Results

**Figure 1:**
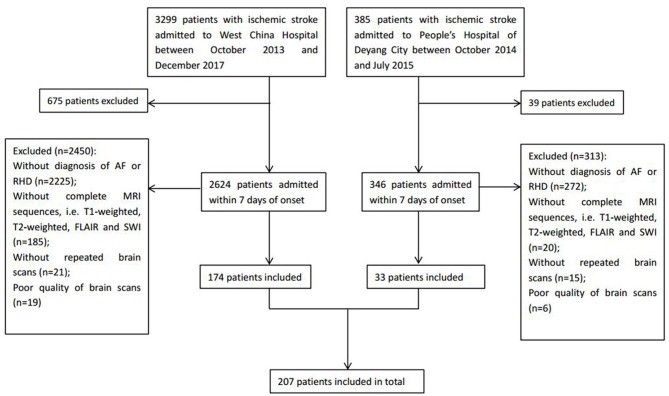
Flowchart of patient enrollment.

**Figure 2:**
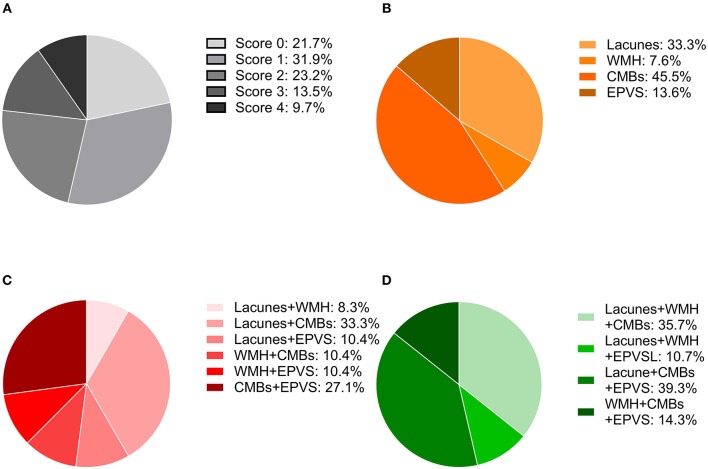
Distribution of the total SVD score and composition of the different scores. **(A)** Frequency of each score in the whole cohort. **(B)** Percentage of each lesion when the SVD score was one. **(C)** Percentages of the combinations of two lesions when the SVD score was two. **(D)** Percentages of the combinations of three lesions when the SVD score was three.

A total of 207 ischemic stroke patients were included in the study (Figure 1), of whom 156 had only AF, 10 only RHD, and 41 both conditions. The patients were aged 67.79 ± 12.84 years, and 58.9% (122/207) were women. According to the etiological classification, 151 (72.9%) patients suffered cardioembolic stroke, 33 (15.9%) atherothrombotic stroke, 5 (2.4%) lacunar stroke, and 18 (8.7%) uncertain stroke. HT occurred in 89 (43.0%) patients, including 64 (30.9%) with HI and 25 (12.1%) with PH. Seventeen (8.2%) patients developed symptomatic HT. Lacunes were present in 91 (44.0%) patients; irregular periventricular WMH (Fazekas score 3) or confluent deep WMH (Fazekas scores 2 or 3) in 56 (27.1%) patients; CMBs in 109 (52.7%) patients; and moderate to severe basal ganglia EPVS (grades 2–4) in 70 (33.8%) patients. In terms of the distribution of the total SVD score, score = 1 was the most common (31.9%), followed by score = 2 (23.2%), score = 0 (21.7%), score = 3 (13.5%), and score = 4 (9.7%) (Figure 2). Figure 2 shows composition of the SVD score for patients with scores 1–3.

Groups of patients with different SVD scores differed significantly in age (*p* < 0.001), sex (*p* = 0.04), prevalence of hypertension history (*p* < 0.001), NIHSS score (*p* = 0.004), systolic pressure (*p* = 0.002), and level of high-density lipoprotein (*p* = 0.01). However, they did not differ significantly in prevalence of diabetes mellitus, hyperlipidemia, smoking, alcohol consumption, use of antithrombotic drugs (antiplatelets and anticoagulants), or thrombolysis (all *p* > 0.05, Supplemental Table 1).

Table 1 shows the differences in the baseline characteristics between patients with and without HT. Patients with HT presented a more severe stroke (*p* < 0.001), a higher level of blood glucose (*p* = 0.004), and lower levels of total cholesterol and low-density lipoprotein (both *p* = 0.02) on admission compared to patients without HT. There was no significant difference in the use of antiplatelet drugs (*p* = 0.27 for prior use, *p* = 0.26 for in-hospital use), while the proportion of patients treated with anticoagulants during hospitalization was lower in the HT group than in the non-HT group (12.4 vs. 45.8%, *p* < 0.001). Thrombolysis was more prevalent in the HT group than in the non-HT group, although the difference was not statistically significant (15.7 vs. 7.6%, *p* = 0.07).

**Table 1:** Differences in baseline characteristics between patients with or without HT.

	**HT (*n* = 89)**	**Non-HT (*n* = 118)**	***p***
Female sex	50 (56.2)	72 (61.0)	0.48
Age, years	68.63 ± 12.91	67.15 ± 12.80	0.41
Hypertension	38 (42.7)	50 (42.4)	0.96
Diabetes mellitus	25 (28.1)	31 (26.3)	0.77
Hyperlipidemia	17 (19.1)	24 (20.3)	0.83
Current or past smoker	20 (22.5)	23 (19.5)	0.60
Current or past drinker	16 (18.0)	21 (17.8)	0.97
Prior use of Antiplatelets	21 (23.6)	36 (30.5)	0.27
Prior use of Anticoagulants	15 (16.9)	19 (16.1)	0.89
Systolic pressure on admission, mmHg	136.02 ± 21.71	135.07 ± 22.83	0.76
Diastolic pressure on admission, mmHg	83.02 ± 16.16	81.47 ± 14.1	0.46
NIHSS score on admission	11 (5–16)	6 (2.75–9.00)	**<** **0.001**
**TREATMENTS IN HOSPITAL**			
Thrombolysis	14 (15.7)	9 (7.6)	0.07
Antiplatelets	70 (78.7)	100 (84.7)	0.26
Anticoagulants	11 (12.4)	54 (45.8)	**<** **0.001**
**LABORATORY TESTS**, **mmol/L**			
Glucose	7.86 ± 2.65	6.85 ± 1.93	**0.004**
Triglyceride	1.23 ± 0.85	1.36 ± 1.20	0.38
Total cholesterol	3.91 ± 0.93	4.22 ± 0.98	**0.02**
High-density lipoprotein	1.35 ± 0.46	1.51 ± 0.97	0.14
Low-density lipoprotein	2.14 ± 0.71	2.39 ± 0.82	**0.02**
**ETIOLOGY OF CURRENT STROKE**			**0.13**
Cardioembolic	70 (78.7)	81 (68.6)	
Atherothrombotic	11 (12.4)	22 (18.6)	
Lacunar	0	5 (4.2)	
Uncertain	8 (9.0)	10 (8.5)	

*Values are n (%), mean ± SD or median (IQR). Boldface indicates statistical significance. HT, hemorrhagic transformation; SD, standard deviation; IQR, interquartile range; NIHSS, National Institutes of Health Stroke Scale*.

**Figure 3:**
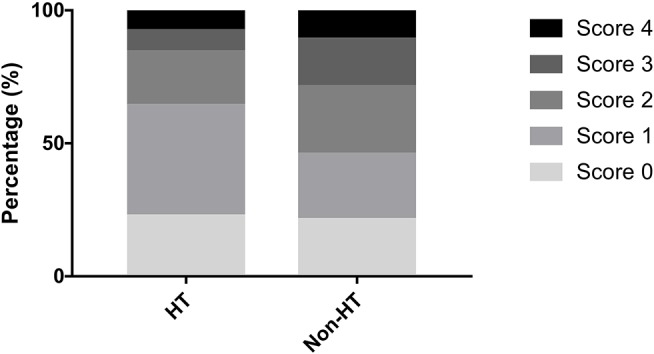
Percentages of different cerebral SVD scores in patients with or without HT.

**Figure 4:**
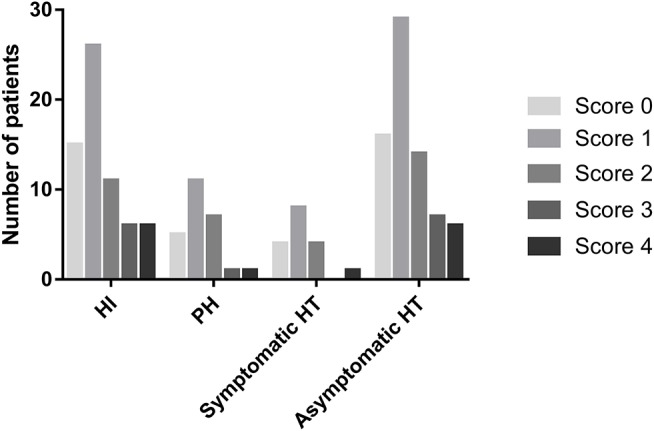
Numbers of patients presenting with different cerebral SVD scores, categorized by the subtypes of HT.

The distribution of the total SVD score was significantly different between patients with HT and those without HT (*p* = 0.04, Figure 3). Patients presenting with only one marker of SVD (score = 1) were more likely to develop HT than those with score≠1 (56.1 vs. 36.9%, *p* = 0.009), while HT was less common in patients with ≥2 points on the SVD score than in those with score <2 (33.3 vs. 51.4%, *p* = 0.009). Regarding the subtypes of HT, SVD score = 1 was always the most common, and there was a decrease in the proportion of patients with HT when the SVD score was higher than one point (Figure 4). After adjusting for age, NIHSS score, blood glucose, total cholesterol, low-density lipoprotein, etiological classification, and treatment during hospitalization with thrombolytic, antiplatelet, and anticoagulant drugs, we found that SVD score = 1 was independently associated with an increased risk of HT (OR, 3.23; 95% CI, 1.48–7.04; *p* = 0.003), while SVD score ≥2 was a protective factor against HT (OR, 0.41; 95% CI, 0.19–0.91; *p* = 0.03; Table 2). The same significant associations were found in the subgroups of patients with HI or asymptomatic HT (all *p* < 0.05, Table 2). A subgroup analysis was conducted on the cohort from West China Hospital that was evaluated with the same 3-T MRI scanner, and the results were similar to those for the whole cohort (Supplemental Table 2).

**Table 2:** Multivariate binary logistic regression to evaluate potential associations between the total SVD score and HT.

	**Total SVD score (per point increasing)**			**SVD score** **=** **1**			**SVD score** **≥2**		
	**OR**	**95% CI**	***p***	**OR**	**95% CI**	***p***	**OR**	**95% CI**	***p***
HT	0.78	0.56–1.07	0.12	**3.23**	**1.48**–**7.04**	**0.003**	**0.41**	**0.19**–**0.91**	**0.03**
HI	0.75	0.55–1.02	0.07	**2.25**	**1.09**–**4.61**	**0.03**	**0.37**	**0.17**–**0.81**	**0.01**
PH	1.06	0.64–1.75	0.83	1.95	0.68–5.60	0.21	1.17	0.34–3.96	0.80
Symptomatic HT	1.19	0.65–2.18	0.58	2.49	0.70–8.91	0.16	0.96	0.22–4.14	0.96
Asymptomatic HT	**0.72**	**0.53**–**0.99**	**0.04**	**2.28**	**1.09**–**4.75**	**0.03**	**0.39**	**0.18**–**0.85**	**0.02**

*Adjusted for age, NIHSS score, blood glucose, total cholesterol, low-density lipoprotein, etiological classification, and treatments after admission (thrombolysis, antiplatelets, and anticoagulants)*.

*Boldface indicates statistical significance*.

*SVD, small vessel disease; OR, odds ratio; CI, confidence interval; HT, hemorrhagic transformation; HI, hemorrhagic infarction; PH, parenchymal hemorrhage*.

We further analyzed the association of each component of the SVD score with HT. When we analyzed each marker on its own, irrespective of the presence of the other three markers, none was significantly associated with HT (all *p* > 0.05, Table 3). Nevertheless, compared to the non-HT group, patients with HT were more likely to present with CMBs only (22.5 vs. 8.5%, *p* = 0.005) and less likely to present with CMBs together with other SVD markers (25.8 vs. 47.5%, *p* = 0.002, Table 3). Similarly, the coexistence of WMHs and other SVD markers was less common in the HT group than in the non-HT group (16.9 vs. 30.5%, *p* = 0.02). After adjusting for age, NIHSS score, blood glucose, total cholesterol, low-density lipoprotein, etiological classification, and treatment during hospitalization with thrombolytic, antiplatelet, and anticoagulant drugs, only CMBs remained significantly associated with HT (CMBs alone: OR, 5.03; 95% CI, 1.78–14.18; *p* = 0.02; coexistence of CMBs and other markers: OR, 0.33; 95% CI, 0.15–0.76; *p* = 0.009).

**Table 3:** Comparison of the components of the SVD score between patients with or without HT.

**SVD marker**	**HT (*n* = 89)**	**Non-HT**	***p***
		**(*n* = 118)**	
Lacunes	35 (39.3)	56 (47.5)	0.24
Lacunes alone	11 (12.4)	11 (9.3)	0.48
Lacune + any of the other three markers	24 (27.0)	45 (38.1)	0.09
WMHs	18 (20.2)	38 (32.2)	0.06
WMHs alone	3 (3.4)	2 (1.7)	0.75
WMHs + any of the other three markers	15 (16.9)	36 (30.5)	**0.02**
CMBs	43 (48.3)	66 (55.9)	0.28
CMBs alone	20 (22.5)	10 (8.5)	**0.005**
CMBs + any of the other three markers	23 (25.8)	56 (47.5)	**0.002**
EPVS	26 (29.2)	44 (37.3)	0.22
EPVS alone	3 (3.4)	6 (5.1)	0.80
EPVS + any of the other three markers	23 (25.8)	38 (32.2)	0.32

*Data are shown as n (%). Boldface indicates statistical significance*.

*CMBs, cerebral microbleeds; EPVS, enlarged perivascular spaces; HT, hemorrhagic transformation; SVD, small vessel disease; WMHs, white matter hyperintensities*.

## Discussion

In the present study, we found that cerebral SVD was common in ischemic stroke patients with AF and/or RHD and that the total SVD score was significantly associated with the occurrence of HT. The association between the total SVD score and HT was not linear. SVD score = 1 correlated with an increased risk of HT, while the presence of two or more MRI markers of SVD (score ≥2) was inversely associated with the occurrence of HT.

About 80% of ischemic stroke patients with AF and/or RHD had cerebral SVD in this study, with CMBs being the most common. Previous studies reported that cerebral SVD was present in ~60% of patients with mild stroke (9) and in about 50% of ischemic stroke patients (28). Both percentages were lower than our observations. However, another study found that 85% of patients with moderate to severe stroke had one or more markers of SVD (29), which was similar to our percentage. Differences in the populations included in the studies may explain the discrepancies between the results. In addition, previous work found that individual markers of SVD were frequently observed in stroke patients (30, 31), and a study in Japan showed that patients with AF had more severe WMHs than those with sinus rhythm (32). Therefore, we speculate that the interaction between ischemic stroke and AF as well as RHD may increase SVD burden, resulting in the relatively high rate of cerebral SVD observed in our cohort.

Although the total SVD score has been suggested to be associated with death and disability after stroke (12, 28, 29), its implications for patients with HT have yet to be investigated. Our findings provide new evidence that the total SVD score plays a non-linear role in the development of HT and its subtypes. Patients in our study were more likely to have HT if they presented with only one marker of SVD. Several studies have reported that a single MRI marker of SVD, such as WMHs or CMBs, is associated with an increased risk of HT (16, 17, 33, 34). Cerebral SVD is associated with increased blood–brain barrier leakage in acute ischemic stroke (35), while HT is believed to result from abnormal permeability of the blood–brain barrier, which allows extravasation of blood products in or out of the ischemic region after restoration of blood flow (36). Therefore, it is plausible that the risk of HT increases with the existence of SVD. Interestingly, we found that the presence of two or more markers of SVD showed a protective effect against HT. As far as we know, the existence of SVD reflects local hypoxia and insufficiency of blood flow, since SVD is characterized by progressive pathological injuries in the brain's small perforating vessels (31). Recently, several studies have analyzed a neuroprotective strategy of ischemic preconditioning to prevent and treat ischemic stroke (37–39). We speculate that the combined presence of different MRI markers of SVD may exert effects like those of ischemic preconditioning, increasing tolerance to vessel fragility and reducing risk of HT. Animal studies are needed to verify this hypothesis.

Interestingly, our results implied that CMBs seemed to accelerate the development of HT, while the presence of other SVD markers may weaken the effect of CMBs on HT and even make CMBs exert protective rather than harmful effects. When we focused on individual markers irrespective of the presence of the other three markers, we found no significant association between each marker and risk of HT. This finding may partly explain why some studies about CMBs reported no relationship with HT (15, 40). Therefore, to better understand the effect of one specific SVD marker on HT, further studies should exclude the presence of other SVD markers, because the interaction between SVD markers may confound the underlying mechanisms.

Our study presents several limitations. First, subjects in this study were ischemic stroke patients with AF and/or RHD, who constitute a relatively specific population and may limit the generalizability of our findings to overall stroke patients. In addition, previous studies (41, 42) indicated that the rate of HT was higher in Asian patients than in Caucasian ones, and therefore, further studies with other subtypes of stroke and additional ethnicities are required to verify our results. Second, this was a retrospective study, so there may be recall bias and missing data in the medical databases. The results of our study should be confirmed in a large, prospective study. Third, both 1.5-T and 3-T MRI scanners were used to assess the markers of SVD, which potentially increased the heterogeneity of SVD detection. Nevertheless, we did verify that the results for the subgroup analyzed on the same 3T scanner were similar to our results for the entire cohort. Studies with the 1.5-T scanner should also be performed to confirm our findings. Fourth, the detection of HT depended on scheduled MRI within 7 days after admission or second CT when neurological deterioration occurred. The sensitivity difference between CT and MRI to detect HT may result in potential biases. Finally, we did not include endovascular treatment as a confounder, which is also a common risk factor for HT (43). Endovascular treatment did not become part of the routine care for acute stroke patients in the local clinical practice during our study period. Therefore, only a small number of patients received this treatment.

## Conclusions

In a cohort of ischemic stroke patients with AF and/or RHD, we investigated, for the first time, the association between the total SVD score and HT. The association between the total SVD score and HT was not linear, since the presence of only one marker of SVD (score = 1) was associated with an increased risk of HT, while the presence of two or more markers of SVD (score ≥2) was a protective factor against HT. These findings suggest a complex role for SVD in the development of HT, and further studies are required to clarify the underlying mechanisms. In addition, our results suggest the need to take into account the total SVD burden, not single SVD markers, when assessing the risk of HT.

## Data Availability

The datasets for this manuscript are not publicly available because the data that support the findings of this study are available from the corresponding author upon reasonable request. Requests to access the datasets should be directed to ML, wyplmh@hotmail.com.

## Ethics Statement

This study was carried out in accordance with the recommendations of the Biomedical Research Ethics Committee of West China Hospital, Sichuan University with written informed consent from all subjects. All subjects gave written informed consent in accordance with the Declaration of Helsinki. The protocol was approved by the Biomedical Research Ethics Committee of West China Hospital, Sichuan University.

## Author Contributions

ML designed and supervised the study. CW, JLiu, and JLi collected the clinical data and analyzed the images. CW performed the statistical analysis and wrote the manuscript draft. JLiu critically revised the manuscript. All authors read and approved the final version submitted for publication.

### Conflict of Interest Statement

The authors declare that the research was conducted in the absence of any commercial or financial relationships that could be construed as a potential conflict of interest.
