# Supplementary material for: A Non-linear Association Between Total Small Vessel Disease Score and Hemorrhagic Transformation After Ischemic Stroke With Atrial Fibrillation and/or Rheumatic Heart Disease
Source: Front Neurol. 2019 Jul 24;10:769. doi: 10.3389/fneur.2019.00769 (PMC6667994; doi:10.3389/fneur.2019.00769)
Supplement: Supplementary file 1 [file Table_1.DOCX]

| Variables | Score 0 (n=45) | Score 1 (n=66) | Score 2 (n=48) | Score 3 (n=28) | Score 4 (n=20) | p |
| --- | --- | --- | --- | --- | --- | --- |
| Female sex, n (%) | 28(62.2) | 43(65.2) | 29(60.4) | 9(32.1) | 13(65.0) | 0.04 |
| Age, years, mean±SD | 61.07±12.64 | 64.76±12.85 | 71.63±11.59 | 74.07±9.54 | 74.90±10.10 | <0.001 |
| Hypertension, n (%) | 12(26.7) | 19(28.8) | 26(54.2) | 17(60.7) | 14(70.0) | <0.001 |
| Diabetes mellitus, n (%) | 10(22.2) | 21(31.8) | 14(29.2) | 8(28.6) | 3(15.0) | 0.57 |
| Hyperlipidemia, n (%) | 7(15.6) | 20(30.3) | 7(14.6) | 5(17.9) | 2(10.0) | 0.13 |
| Current or past smoker, n (%) | 11(24.4) | 14(21.2) | 8(16.7) | 9(32.1) | 1(5.0) | 0.19 |
| Current or past drinker, n (%) | 11(24.4) | 13(19.7) | 7(14.6) | 5(17.9) | 1(5.0) | 0.40 |
| Antiplatelets before admission, n (%) | 9(20.0) | 15(22.7) | 15(31.3) | 9(32.1) | 9(45.0) | 0.22 |
| Anticoagulants before admission, n (%) | 6(13.3) | 12(18.2) | 12(25.0) | 4(14.3) | 0 | 0.13 |
| Systolic pressure on admission, mmHg, mean±SD | 125.04±20.08 | 135.00±19.51 | 142.69±23.92 | 136.79±18.96 | 141.40±28.31 | 0.002 |
| Diatolic pressure on admission, mmHg, mean±SD | 77.07±14.72 | 82.62±15.18 | 84.00±14.00 | 84.21±15.42 | 84.60±15.68 | 0.13 |
| NIHSS score on admission, median (IQR) | 9(5-16) | 9(4-15) | 5(3-12.5) | 5.5(1.5-7.75) | 5.5(3-10) | 0.004 |
| Treatments in hospital, n (%) |  |  |  |  |  |  |
| Thrombolysis | 8(17.8) | 9(13.6) | 4(8.3) | 2(7.1) | 0 | 0.22 |
| Antiplatelets | 38(84.4) | 53(80.3) | 37(77.1) | 25(89.3) | 17(85.0) | 0.69 |
| Anticoagulants | 14(31.1) | 20(30.3) | 18(37.5) | 8(28.6) | 5(25.0) | 0.85 |
| Laboratory test, mmol/L, mean±SD |  |  |  |  |  |  |
| Glucose | 7.07±1.38 | 7.53±2.57 | 7.32±2.95 | 6.89±1.67 | 7.37±2.14 | 0.76 |
| Triglyceride | 1.24±0.77 | 1.43±1.25 | 1.2±0.79 | 1.15±1.18 | 1.54±1.37 | 0.57 |
| Total chlesterol | 4.1±0.91 | 4.14±1.05 | 4.09±1.03 | 4.13±0.86 | 3.83±0.92 | 0.82 |
| High-density lipoprotein | 1.37±0.34 | 1.37±0.43 | 1.44±0.51 | 1.91±1.83 | 1.18±0.38 | 0.01 |
| Low-density lipoprotein | 2.3±0.78 | 2.33±0.82 | 2.23±0.77 | 2.26±0.72 | 2.22±0.84 | 0.96 |

**Supplemental table 1** Comparison of baseline characteristics among patients with different small vessel disease scores

Abbreviations: NIHSS, National Institutes of Health Stroke Scale; SD, standard deviation; IQR, interquartile range.

**Supplemental table 2** Association between the total SVD score and HT in patients from West China Hospital

|  | Total SVD score (per score increasing) | | | SVD score=1 | | | SVD score≧2 | | |
| --- | --- | --- | --- | --- | --- | --- | --- | --- | --- |
|  | OR | 95% CI | p | OR | 95% CI | p | OR | 95% CI | p |
| HT | 0.77 | 0.55-1.09 | 0.14 | **3.46** | **1.52-7.89** | **0.003** | **0.32** | **0.13-0.77** | **0.01** |
| HI | 0.77 | 0.55-1.07 | 0.12 | **2.26** | **1.05-4.88** | **0.04** | **0.34** | **0.15-0.81** | **0.01** |
| PH | 1.03 | 0.57-1.84 | 0.94 | 2.11 | 0.63-7.05 | 0.22 | 0.86 | 0.18-4.15 | 0.85 |
| Symptomatic HT | 1.34 | 0.64-2.81 | 0.44 | 2.79 | 0.62-12.51 | 0.18 | 0.72 | 0.09-5.89 | 0.75 |
| Asymptomatic HT | 0.70 | 0.50-1.00 | 0.05 | **2.34** | **1.07-5.10** | **0.03** | **0.32** | **0.13-0.77** | **0.01** |

Adjusted for age, National Institutes of Health Stroke Scale score, blood glucose, total cholesterol, low-density lipoprotein, etiology of stroke, and treatments after admission (thrombolysis, antiplatelets, and anticoagulants)

Boldface indicates statistical significance.

Abbreviations: SVD, small vessel disease; OR, odds ratio; CI, confidence interval; HT, hemorrhagic transformation; HI, hemorrhagic infarction; PH, parenchymal hemorrhage.
